# The Treatment of COVID-19 With Monoclonal Antibody Therapy: Patient-Reported Outcomes

**DOI:** 10.7759/cureus.29247

**Published:** 2022-09-16

**Authors:** Daniella Lamour, Nika Vafadari, Lisa M Clayton, Joshua J Solano, Patrick G Hughes, Richard D Shih, Scott M Alter

**Affiliations:** 1 Department of Emergency Medicine, Florida Atlantic University Charles E. Schmidt College of Medicine, Boca Raton, USA

**Keywords:** covid-19 treatment, public health, emergency medicine, monoclonal antibodies, covid-19

## Abstract

Objective

There have been many efforts to research and produce treatment modalities for COVID-19. Monoclonal antibodies have been one of the effective treatments since their approval by the US Food and Drug Administration (FDA) under emergency use authorization (EUA) in 2020. This study surveyed COVID-19 patients about their disease course and experience with monoclonal antibody treatment.

Methods

Patients who received monoclonal antibody treatment between February 12, 2021, and June 2, 2021, at a South Florida community hospital were enrolled in the study. This included patients over 18 years of age with a confirmed positive COVID-19 test result, with mild to moderate symptoms within 10 days of onset and identified as high risk for progression to severe disease. There were no exclusion criteria. After 30 days, patients were followed up via a structured telephone survey regarding subsequent emergency department (ED) visits for worsening COVID-19 symptoms, need for oxygenation, intubation, and death. Secondary outcomes were adverse effects and patient perceptions.

Results

Among the 119 patients who received monoclonal antibodies during the established time frame, 93 (78.1%) consented to participate in the telephone survey. Of these, 11.8% had a subsequent visit to the ED for worsening COVID-19 symptoms, 6.5% required oxygen, and 2.2% were admitted to the intensive care unit (ICU). There were no reported intubations or deaths. The vast majority (91.4%) would recommend monoclonal antibody treatment to others.

Conclusion

Patients who received monoclonal antibody therapy had low rates of subsequent ED visits and rarely required oxygen or ICU admission. The majority of patients would recommend treatment with monoclonal antibodies to others.

## Introduction

COVID-19, caused by SARS-CoV-2, is a devastating disease that mainly targets the human respiratory tract. Rapid transmission of the enveloped, single-stranded RNA virus via air droplets has since caused a catastrophic pandemic affecting over 120 million people worldwide with more than two and a half million deaths [1]. Patient presentation may vary anywhere from asymptomatic to severe symptoms of pneumonia, acute respiratory distress syndrome, or multi-organ failure [2]. With the variety of respiratory and extrapulmonary symptoms, as well as the multi-organ nature of the disease, management guidelines have yet to be established, as data regarding treatment options are constantly evolving.

Immunotherapy has proven to be effective against past viruses, including severe acute respiratory syndrome (SARS), Ebola, and influenza [3]. Monoclonal antibodies are a form of passive immunotherapy that may serve as an effective therapeutic intervention against infectious entities [3]. There is a plethora of investigational neutralizing monoclonal antibodies being studied as a potential treatment option for SARS-CoV-2 [4]. They bind to the receptor-binding domain of the spike protein of the COVID-19 strand, preventing attachment and thus entry into the human angiotensin-converting enzyme 2 (ACE2) receptor [5]. The expression of ACE2 receptors on various human organs highlights the multisystem nature of the disease. While studies have detected the highest levels of the virus in the respiratory tract of SARS-CoV-2-infected patients, lower levels have also been detected in the blood, liver, heart, brain, and kidneys [3].

Although its effectiveness continues to be evaluated with evolving SARS-CoV-2 variants, bamlanivimab (BAM) was first granted emergency use authorization (EUA) in November 2020 for the treatment of mild to moderate COVID-19 in adults and pediatric patients (12 years of age and older weighing at least 40 kg) with positive results of direct SARS-CoV-2 viral testing and who are at high risk for progressing to severe COVID-19 and/or hospitalization [6]. The support for its use was predicated on an interim analysis of the BLAZE-1 trial, a double-blinded randomized controlled trial (RCT), which illustrated a reduction in emergency department (ED) visits and hospitalizations in mild to moderate cases. However, in the final analysis of the BLAZE-1 trial, bamlanivimab monotherapy did not have a significant difference in reducing the viral load but was effective in reducing the viral load in combination with etesevimab in mild to moderate COVID-19 illness [4].

After the final publication, the US Food and Drug Administration (FDA) issued another emergency use authorization in February 2021 for dual therapy with bamlanivimab and etesevimab [7]. After the emergency use authorization took effect, bamlanivimab alone was associated with a reduction of hospitalizations and symptom deterioration in a population at high risk for severe disease and solid organ transplant patients [8]. On April 12, 2021, the phase III REGEN-COV 2069 clinical trial showed that casirivimab and imdevimab reduced the risk of symptomatic COVID-19 infections by 81% [9]. While there are quite a few clinical trials highlighting their effectiveness, there are not many articles addressing the patients’ outlook on monoclonal antibody treatments.

Clinicians around the world are faced with rapidly changing treatment options for COVID-19; hence, having insight from patients receiving these monoclonal antibodies adds additional information for its further use. This study describes the outcomes and perceptions of patients diagnosed with COVID-19 who have received outpatient treatment with monoclonal antibodies including bamlanivimab (BAM), bamlanivimab/etesevimab (BAM-E), and casirivimab/imdevimab (REGEN-COV).

## Materials and methods

Study design and participants

This is a survey of patients who received outpatient monoclonal antibodies for the treatment of COVID-19 at a community hospital in South Florida. The study was approved by the institutional review boards of the hospital and its affiliated university. Patients over 18 years of age with a confirmed positive COVID-19 test result, with mild to moderate symptoms within 10 days of onset, and identified as high risk for progression to severe disease were referred to the hospital to receive the monoclonal antibodies. Per the US National Institutes of Health’s COVID-19 treatment guidelines, mild illness was defined as individuals experiencing symptoms without dyspnea or abnormal chest imaging, while moderate illness was defined as individuals with evidence of lower respiratory disease but with saturations at or above 94% [10]. All patients who received monoclonal antibody treatment from February 12, 2021, to June 2, 2021, were included in the study. There were no exclusion criteria.

Patients were prescheduled for the monoclonal antibody infusion in the ED and overseen by emergency physicians. From February 12 to March 18, 2021, patients received bamlanivimab monotherapy. On March 19, 2021, the hospital switched its treatment protocol to bamlanivimab plus etesevimab. On April 28, 2021, the hospital again switched its treatment protocol to casirivimab plus imdevimab. The hospital only offered one monoclonal antibody treatment protocol at any given time.

Data collection

Electronic medical records were queried by the hospital information technology/medical record department for COVID-19 patients treated with monoclonal antibodies. Study investigators then performed a chart review of identified patients. Variables extracted from the ED chart included patient demographics, past medical history, duration of COVID-19 symptoms, time from COVID-19 diagnosis to monoclonal antibody treatment, and which monoclonal antibody was administered.

On or after 30 days from monoclonal antibody treatment, one of the study investigators called each patient to perform a structured telephone survey and assess outcomes. Questions asked included initial COVID-19 symptoms, side effects of monoclonal antibody treatment, subjective improvement after treatment, ED visits before or after treatment, oxygen requirement, hospital admission, intensive care unit (ICU) admission, intubation, mortality, days to symptom resolution, positive recommendation of treatment, and referral source of treatment (see Appendix for survey questions).

Outcomes

The primary outcomes of this study were subsequent ED visits, oxygen requirement, hospitalization, and death among patients who received monoclonal antibody therapy for the treatment of COVID-19. Furthermore, this study evaluated patient experience with and side effects of monoclonal antibody therapy. Patients were grouped and analyzed by monoclonal antibody treatment received. Descriptive statistics were performed using SPSS version 27.0 (IBM SPSS Statistics for Windows, Armonk, NY).

## Results

Characteristics of study subjects

Monoclonal antibody therapy for the treatment of COVID-19 was administered to 119 patients who were included in the study. Ninety-seven (81.5%) patients were able to be contacted for the telephone survey, and 93 (78.2%) consented to participate. Baseline characteristics were mostly similar among those who completed the survey and those who did not (Table 1).

**Table 1:** Background characteristics by completion of survey BAM: bamlanivimab; BAM-E: bamlanivimab/etesevimab; BMI: body mass index; COPD: chronic obstructive pulmonary disease; REGEN-COV: casirivimab/imdevimab

	No survey (n=26)	Survey (n=93)
Age, mean years (SD)	63.0 (16.2)	60.2 (14.7)
Female gender, n (%)	7 (26.9)	53 (57.0)
BMI, mean kg/m^2^ (SD)	30.3 (9.7)	32.7 (9.7)
Race, n (%)		
White	20 (76.9)	68 (73.1)
Black	2 (7.7)	11 (11.8)
Asian	1 (3.8)	0 (0)
Native American	0 (0)	0 (0)
Pacific Islander	0 (0)	0 (0)
Others	3 (11.5)	14 (15.1)
Ethnicity, n (%)		
Not Hispanic	22 (84.6)	84 (90.3)
Hispanic	3 (11.5)	4 (4.3)
Others	1 (3.8)	5 (5.4)
Medical history, n (%)		
Hypertension	10 (38.5)	39 (41.9)
Diabetes	8 (30.8)	14 (15.1)
Cardiovascular disease	4 (15.4)	19 (20.4)
COPD	1 (3.8)	7 (7.5)
Immunosuppressive disease	4 (15.4)	13 (14.0)
Chronic kidney disease	1 (3.8)	2 (2.2)
None	9 (34.6)	30 (32.3)
Symptomatic days to treatment, mean days (SD)	6.0 (2.8)	6.1 (2.2)
Days from COVID-19 diagnosis to treatment, mean days (SD)	3.3 (2.0)	3.7 (2.3)
Monoclonal antibody treatment, n (%)		
BAM	8 (30.8)	42 (45.2)
BAM-E	12 (46.2)	35 (37.6)
REGEN-COV	6 (23.1)	16 (17.2)

Main results

The most common symptoms of COVID-19 prior to monoclonal antibody infusion were fever/chills, fatigue, cough, and body aches (Table 2). After the infusion, 76.3% of patients had no side effects. Of the reported side effects, fever was the most common in 10.8% of patients, with difficulty breathing next in 7.5% of participants (Table 3).

**Table 2:** Symptoms prior to monoclonal antibody treatment BAM: bamlanivimab; BAM-E: bamlanivimab/etesevimab; REGEN-COV: casirivimab/imdevimab

Symptoms	BAM (n=42)	BAM-E (n=35)	REGEN-COV (n=16)	All surveyed patients (n=93)
Shortness of breath	16 (38.1%)	11 (31.4%)	7 (43.8%)	34 (36.6%)
Fever/chills	29 (69.0%)	22 (62.9%)	13 (81.3%)	64 (68.8%)
Cough	21 (50.0%)	25 (71.4%)	9 (56.3%)	55 (59.1%)
Fatigue	26 (61.9%)	24 (68.6%)	12 (75.0%)	62 (66.7%)
Body aches	18 (42.9%)	17 (48.6%)	11 (68.8%)	46 (49.5%)
Headache	19 (45.2%)	9 (25.7%)	5 (31.3%)	33 (35.5%)
Loss of taste/smell	11 (26.2%)	13 (37.1%)	4 (25.0%)	28 (30.1%)
Sore throat	13 (31.0%)	9 (25.7%)	0 (0%)	22 (23.7%)
Congestion/runny nose	10 (23.8%)	12 (34.3%)	1 (6.3%)	23 (24.7%)
Nausea/vomiting	7 (16.7%)	5 (14.3%)	6 (37.5%)	18 (19.4%)
Diarrhea	5 (11.9%)	7 (20.0%)	1 (6.3%)	13 (14.0%)

**Table 3:** Side effects of monoclonal antibody treatment BAM: bamlanivimab; BAM-E: bamlanivimab/etesevimab; REGEN-COV: casirivimab/imdevimab

Side effects	BAM (n=42)	BAM-E (n=35)	REGEN-COV (n=16)	All surveyed patients (n=93)
Fever	4 (9.5%)	5 (14.3%)	1 (6.3%)	10 (10.8%)
Pain	0 (0%)	0 (0%)	0 (0%)	0 (0%)
Rash	0 (0%)	0 (0%)	0 (0%)	0 (0%)
Difficulty breathing	6 (14.3%)	1 (2.9%)	0 (0%)	7 (7.5%)
Headache	3 (7.1%)	1 (2.9%)	0 (0%)	4 (4.3%)
Nausea/vomiting	2 (4.8%)	2 (5.7%)	0 (0%)	4 (4.3%)
Weakness	2 (4.8%)	3 (8.6%)	0 (0%)	5 (5.4%)
Others	4 (9.5%)	1 (2.9%)	0 (0%)	5 (5.4%)
None	30 (71.4%)	26 (74.3%)	15 (93.8%)	71 (76.3%)

Of the patients, 11.8% had a subsequent ED visit for worsening COVID-19 sequela (Table 4). Of those, six (6.5%) required oxygen, five (5.4%) required hospital admission, and two (2.2%) were admitted to the ICU. There were no intubations or deaths reported. Subjective improvement was reported in 67.7% of patients. The average time for symptom resolution was 15.6 days (SD 12.9).

**Table 4:** COVID-19 course BAM: bamlanivimab; BAM-E: bamlanivimab/etesevimab; ED: emergency department; ICU: intensive care unit; REGEN-COV: casirivimab/imdevimab

Outcomes	BAM (n=42)	BAM-E (n=35)	REGEN-COV (n=16)	All surveyed patients (n=93)
ED visit before infusion	10 (23.8%)	11 (31.4%)	8 (50.0%)	29 (31.2%)
Subsequent ED visit within one month	8 (19.0%)	3 (8.6%)	0 (0%)	11 (11.8%)
ED visit for COVID-19 sequela	8 (19.0%)	3 (8.6%)	0 (0%)	11 (11.8%)
Oxygen	4 (9.5%)	1 (2.9%)	1 (6.3%)	6 (6.5%)
Hospital admission	3 (7.1%)	2 (5.7%)	0 (0%)	5 (5.4%)
Hospital length of stay, mean days (SD)	6.0 (2.0)	4.0 (2.8)	0 (0)	5.2 (2.3)
ICU admission	2 (4.8%)	0 (0%)	0 (0%)	2 (2.2%)
Intubated	0 (0%)	0 (0%)	0 (0%)	0 (0%)
Death	0 (0%)	0 (0%)	0 (0%)	0 (0%)
Subjective improvement after treatment	24 (57.1%)	25 (71.4%)	14 (87.5%)	63 (67.7%)
Symptom resolution, mean days (SD)	19.0 (15.0)	12.6 (8.6)	13.4 (13.1)	15.6 (12.9)
Positive recommendation of treatment	40 (95.2%)	30 (85.7%)	15 (93.8%)	85 (91.4%)

The majority of participants (91.4%) recommended the monoclonal antibody infusion to others. The most common way participants found out about monoclonal antibody treatments was by various types of physician referrals (Table 5).

**Table 5:** How patients heard about monoclonal antibody treatment BAM: bamlanivimab; BAM-E: bamlanivimab/etesevimab; ED: emergency department; REGEN-COV: casirivimab/imdevimab

	BAM (n=42)	BAM-E (n=35)	REGEN-COV (n=16)	All surveyed patients (n=93)
Referral from treatment ED or hospital personnel	7 (16.7%)	4 (11.4%)	8 (50.0%)	19 (20.4%)
Referral from outside hospital ED or hospital personnel	7 (16.7%)	3 (8.6%)	1 (6.3%)	11 (11.8%)
Referral from primary care physician	8 (19.0%)	11 (31.4%)	2 (12.5%)	21 (22.6%)
Referral from other physicians	13 (31.0%)	8 (22.9%)	3 (18.8%)	24 (25.8%)
Friend or family member	6 (14.3%)	9 (25.7%)	2 (12.5%)	17 (18.3%)
Media outlet	1 (2.4%)	0 (0%)	0 (0%)	1 (1.1%)

## Discussion

In this study of patients who received monoclonal antibodies for the treatment of COVID-19, the primary outcomes of subsequent ED visit, oxygen requirement, intubation, or death reported were minimal. Of the few subsequent ED visits for worsening COVID-19 symptoms, while oxygen was necessary for half of the patients, there were no intubations or deaths. Symptom resolutions averaged 15.6 days. Although only 65% of patients reported subjective improvement in symptoms after the treatment, over 90% recommend treatment to others. These results support a positive outlook for monoclonal antibody treatments. Further, the majority of patients reported no side effects.

Although statistical significance cannot be drawn from this study, monoclonal antibody therapy has been proven effective in several other randomized controlled trials (RCTs) in reducing oxygen requirement, hospitalization, and death. The results of those RCTs were similar to those found in this study. In addition, there are currently over 10 monoclonal antibodies being researched potentially allowing for future treatment options for any COVID-19 variants that may arise.

An important limitation of this study is that the predominant circulating COVID-19 variant changed during the duration of the study, which was initially alpha and later delta. There has been variable susceptibility of the monoclonal antibodies to each variant. As a result of the evolving variants, the FDA has adjusted emergency use authorization (EUA) recommendations on several occasions [11]. In January 2022, the FDA revoked the emergency use authorizations for bamlanivimab/etesevimab and casirivimab/imdevimab. Sotrovimab, another monoclonal antibody that had also been under FDA EUA, was not given at the study site during the study period.

This study has several limitations. First, the study only included patients who received bamlanivimab, bamlanivimab/etesevimab, and casirivimab/imdevimab. The monoclonal antibody treatments are continuously changing, and the number of COVID-19 variants is increasing, thus limiting the generalizability of the findings to current or future COVID-19 strains. Second, the survey response rate was 78.2%, with 3.3% not providing consent and 18.5% unable to be reached. Although the overall response rate is high for a phone survey, bias may still be introduced given the unknown outcomes of the non-respondents, which can be expected when conducting telephone interviews. Some patients expressed their concern that the hospital may have been calling regarding a financial bill for the monoclonal antibody infusion, which may have resulted in this loss to follow-up. Third, patients from outside of the hospital’s normal catchment area sought monoclonal antibody therapy at the hospital. Thus, if patients needed to seek emergency care, they might not have returned to the study hospital.

## Conclusions

In summary, patients who received monoclonal antibody therapy had low rates of subsequent ED visits and rarely required oxygen or ICU admission. This is consistent with prior studies of patients with mild to moderate disease. Additionally, the majority of patients would recommend treatment with monoclonal antibodies to others. With rising rates of COVID-19 and the increased resistance and hesitancy to receive vaccination, there continues to be a role for monoclonal antibody therapy among adults with high risk of developing worsening COVID-19. Further studies are needed with current COVID-19 variants to establish the generalizability of these therapies.

## Appendices

Telephone survey questions

1. What were your initial COVID-19 symptoms before receiving the monoclonal antibody infusion? Fever or chills; cough; shortness of breath or difficulty breathing; fatigue; muscle or body aches; headache; new loss of taste or smell; sore throat; congestion or runny nose; nausea or vomiting.

2. Did you go to the emergency department for COVID-19-related symptoms before receiving the monoclonal antibody infusion?

3. How did you hear about monoclonal antibody therapy? Referral from the emergency department or hospital personnel; referral from outside hospital emergency department or hospital personnel; referral from primary care physician; referral from other physicians; friend or family member; media outlet; others.

4. Did you physically feel an improvement of symptoms after the monoclonal antibody infusion?

5. Do you experience any of the following side effects of the infusion? Fever; pain; rash; difficulty breathing; headache; nausea or vomiting; weakness; others.

6. Did you need oxygen within one month after the infusion?

7. Did you have to go to the ED within a month of the infusion? If yes, was it for worsening coronavirus symptoms? Was it for something unrelated to COVID-19? If yes, why did you go into the emergency room (ER)? Did you have to be admitted to the hospital? If yes, were you admitted to the ICU? Were you intubated? How long were you hospitalized?

8. When did your COVID-19 symptoms fully resolve?

9. Would you recommend others to receive the monoclonal antibody infusion?
